# Surgical Intervention for Spastic Upper Extremity Improves Lower Extremity Kinematics in Spastic Adults: A Collection of Case Studies

**DOI:** 10.3389/fbioe.2020.00116

**Published:** 2020-02-21

**Authors:** Nojoud AlHakeem, Elizabeth Anne Ouellette, Francesco Travascio, Shihab Asfour

**Affiliations:** ^1^Biomechanics Research Laboratory, Department of Industrial Engineering, University of Miami, Coral Gables, FL, United States; ^2^Miami Orthopedics & Sports Medicine Institute, Baptist Health South Florida, Coral Gables, FL, United States; ^3^Department of Orthopedics, Herbert Wertheim College of Medicine, Florida International University, Miami, FL, United States; ^4^Musculoskeletal Biomechanics Laboratory, University of Miami, Coral Gables, FL, United States; ^5^Mount Sinai Medical Center, Max Biedermann Institute for Biomechanics, Miami Beach, FL, United States; ^6^Department of Orthopaedic Surgery, Miller School of Medicine, University of Miami, Miami, FL, United States

**Keywords:** cerebral palsy, gait analysis, motion capturing, stroke, ulnar nerve

## Abstract

**Background:**

Spasticity of the upper extremity often occurs after injury to the upper motor neurons (UMN). This condition can greatly interfere with the hand positioning in space and the functional use of the arm, affecting many daily living activities including walking. As gait and balance involve the coordination of all segments of the body, the control of upper limbs movement is necessary for smooth motion and stability. The purpose of this study was to assess the effects of surgical interventions on upper extremity spasticity to gait patterns in three spastic patients, as a way to assess the effect on patient’s mobility.

**Methods:**

Three patients with an anoxic brain injury, upper extremity spasticity, and an altered gait participated in this study. A specific treatment plan based on the patient was tailored by the orthopedic hand surgeon to help release the contractures and spastic muscles. Three-dimensional gait analysis was performed before surgery, 3, 6, and 12 months postoperatively. During each experimental session, the patient walked at a self-selected pace in a straight line across four force plates embedded into the floor (Kistler^®^). Motion data were acquired using Vicon^®^ Motion Capturing System. Spatiotemporal measurements as well as bilateral kinematics of the hip, knee and ankle were studied. The results from matched non-disabled controls were included as reference.

**Results:**

Overtime, clinical assessment displayed recovery in hand functions and restored sensation in the fingers. Gait analysis results demonstrated overall improvements in spatiotemporal parameters, specifically in cadence and walking speed. Improvements in kinematics of the lower limbs were also evident.

**Conclusion:**

The results of this study indicated that, within a timeframe of one year, gait patterns improved in all patients. These observations suggest that, over time, upper limb surgery has the potential to improve the biomechanics of gait in spastic patients.

## Introduction

Impairments of motor and sensory function and coordination are often consequences of injury to the central nervous system (Thevenin-Lemoine et al., 2013; Photopoulos et al., 2014). Upper motor neuron syndrome (UMNS) is a condition that arises as a result of the disturbance of the UMN inhibitory pathways extending all the way from the cerebral cortex to the lower end of the spinal cord (Sheean, 2002). Damage to these pathways lead to symptoms including weakness, spasticity and clonus (Emos and Agarwal, 2018). Clinically, spasticity has been defined as an increased velocity-dependent lengthening in tonic stretch reflexes with exaggerated tendon jerks resulting from hyperexcitability of the stretch reflex (Lance, 1980). Spasticity is often one of the most troublesome components of UMN injury (Bose et al., 2015), and can lead to abnormal postures and joint contractures in the long term (Emos and Agarwal, 2018). Spasticity is also secondary to cerebrovascular accidents and traumatic brain injury (Porretta, 2016). Within these conditions, it affects 17–38% (Sommerfeld et al., 2004; Lundström et al., 2008) of patients with stroke and as many as 77% of patients with cerebral palsy (Christensen et al., 2008). The acute manifestations of UMNS tend to be most severe in the arms and legs (Purves et al., 2001), developing contractures and painful limb deformities. The literature describes a number of common patterns of UMN dysfunction that are generated by combinations of voluntary and involuntary motor behaviors which produces stereotypic movement patterns, such as the flexed elbow, the clenched fist, the flexed hip, and the stiff knee (Mayer et al., 1997, 2001; Mayer and Esquenazi, 2003; Esquenazi and Mayer, 2004; Esquenazi et al., 2013).

Human walking requires specific coordination patterns between upper and lower body limbs to ambulate safely and efficiently. Studies agree that the ordinary reciprocally phased arm-swing during human locomotion plays an active role in the postural control of the body thus increasing the efficiency of gait (Jackson et al., 1983; Eke-Okoro et al., 1997), therefore, any deliberate changes of the arm movement during gait will influence gait parameters hence changing gait efficiency (Ford et al., 2007).

Treatment interventions for upper and lower limbs are performed separately with the sole purpose of relieving pain or correcting deformities caused by spasticity (Tracy, 1976; Waters et al., 1977; Roper et al., 1978; Waters, 1978; Bell et al., 2002; Carlson et al., 2009; Marciniak, 2011). Multiple studies have looked at the association between botulinum toxin type A (BTXA) injection into the spastic arm and gait changes in adults (Wilson et al., 1997; Hesse et al., 1998; Bakheit and Sawyer, 2002; Hirsch et al., 2005; Esquenazi et al., 2008). Improvements in the ROM of the ankle and knee as well as stride time and walking speed were reported, however, the treatment was not always successful and the effects were often only transient. When the deformity is not permanent it can be treated by repeated botulinum toxin injections. If there are contractures, surgical intervention may be indicated (Thevenin-Lemoine et al., 2013). To date, the effect of surgical intervention of spastic upper limbs on the biomechanics of gait is unknown.

We hypothesized that surgical intervention of a spastic upper limb will improve gait patterns in both the spastic and the sound side. The purpose of this study was to assess the effects of surgical intervention to upper limbs on gait pattern in spastic patients with gait abnormalities. To provide a quantitative explanation of how upper limb surgical intervention affects mobilization in spastic patients, gait parameters and lower limbs joint kinematics were assessed.

## Methods

### Patients

Three patients with an anoxic brain lesion (two stroke patients SP1, SP2, and one CP patient SP3) were recruited through the hand surgeon’s clinic. The criteria for eligibility and inclusion in the study were (Thevenin-Lemoine et al., 2013) upper limb spasticity due to an anoxic brain lesion, Photopoulos et al. (2014) observed gait abnormalities, Sheean (2002) an ability to ambulate independently with or without assistive devices, and (Emos and Agarwal, 2018) no history of previous surgery to release spasticity in the upper limb. In all three patients, there were no pharmacological treatment to reduce spasticity and no history of rehabilitation on upper or lower limbs within 6 months prior to enrollment in the study. Three normal non-disabled subjects were recruited to serve as gender, age (within ±11 years), height (within ±7 cm), weight (within ±12 kg) matched controls for the spastic patients. The non-disabled controls had no history of musculoskeletal injury and no apparent gait abnormalities.

The research protocol was reviewed and approved by our Institutional Research Review Committee and all participants gave informed consent to participate in the study.

#### SP1

SP1 was a 73-year-old (height 152 cm, weight 93 kg, and BMI 40.3) right hand dominant male with right side spasticity 24 months post stroke, causing contracture and spastic flexion deformity of the right elbow and affecting the right hand at the little and ring fingers. He presented to the clinic with slight pain in the right elbow and progressive contraction of the small and ring finger. Based on clinical examination he was also assessed with hypertension, right side carpal tunnel syndrome, and right side ulnar nerve entrapment at the elbow. In addition, impairment of gait was also visually observed by the surgeon as the patient was in a wheel chair and had limited mobility. His medical history showed that no prior interventions were done to improve right side spasticity. Preoperative physical examination results confirmed that the patient had spasticity in right hand intrinsic, finger flexors, and elbow flexors. Electromyography (EMG) results showed that the patient had bilateral carpel tunnel but no ulnar nerve entrapment. Additionally, Semmes Weinstein (SW) sensory exam results showed loss of sensation in all right fingers. The patient wished to proceed with surgical release in an attempt to improve the right side upper extremity function and was scheduled for surgery. Based on the diagnosis, the procedures performed were right flexor slide at the elbow with ulnar nerve and carpal tunnel releases. Preoperative gait analysis assessment demonstrated reduced cadence, stride length and single support phase, as well as slower walking speed compared to the matched control. A decrease in knee flexion was also evident on the affected and sound side.

#### SP2

SP2 was a 52-year-old (height 164 cm, weight 60 kg, and BMI 22.3) right hand dominant female with left side spasticity 60 months post stroke that affected her left hand, wrist, elbow, and shoulder. The patient has a history of migraine, seizures, and depression. She presented to the clinic complaining of severe tightness in her left wrist, elbow, and shoulder preventing her from being able to use her left hand. Gait impairment was also visually observed by the surgeon. Her medical history included only BTXA injections on the left arm. Preoperative physical examination confirmed left hand spasticity in finger flexors and pronator teres. EMG with nerve conduction study was normal. SW sensory exams results showed diminished sensation in the left index, long and thumb fingers. However, the ring and small fingers showed complete loss of sensation. Based on the preoperative diagnosis, the surgery performed was left elbow flexor slide with release of the ulnar nerve. Preoperative gait analysis findings showed that the patient had reduced cadence and single support phase, as well as slower walking speed compared to the matched control. Similarly, there was a noticeable decrease in hip and knee flexion on both the affected and sound side.

#### SP3

SP3 was a 20-year-old (height 171 cm, weight 55 kg, and BMI 18.8) left hand dominant male suffering from right side spasticity affecting the elbow, forearm, wrist and fingers due to CP. He presented to the clinic complaining from right upper extremity loss of function and range of motion (ROM), with flexion of the elbow. His medical history included physical therapy treatments to help with the hemiparesis and spasticity with no recollection of success. Preoperative physical assessment showed that there was slight clawing of the right hand fingers and a marked weakness of the right hand where the patient could not abduct his right thumb. There was also a slight flexion in the right wrist and a slight degree of increased tone in the right elbow. However, the ROM in his right shoulder was normal. EMG results confirmed right hand carpal tunnel. SW sensory exam results were normal in all right fingers. The patient was scheduled for surgery in attempt to improve the functions in his right arm. The procedures performed were flexor slide at the right elbow with ulnar nerve and carpal tunnel releases. Preoperative gait analysis demonstrated reduced cadence and single support phase, as well as slower walking speed compared to the matched control. Additionally, an increase in hip flexion and a decrease of knee flexion was evident on both the affected and sound side. It was also observed that the ankle on the affected side remained in dorsiflexion throughout gait.

### Surgical Techniques

Wrist flexion deformity and ulnar nerve entrapment was a common occurrence in all. Therefore, flexor slide and ulnar nerve release were performed in all patients. Flexor slide was performed through medial elbow approach with releases of ulnar nerve, flexor origin on medial epicondyle and ulnar attachment (Waters, 1978; Dellon, 1991; Patterson et al., 2000). This created a shortening of 2–3 inches in the flexor origin. Carpal tunnel was found in two patients and were performed using the endoscopic Chow technique (Chow and Hantes, 2002). All patients were first casted in extension for 6 weeks then splinted in extension for another 6 weeks with motion. Moreover, the only form of rehabilitation all three patients received was occupational therapy post-surgery on the affected hand.

### Gait Analysis

Gait abnormalities were visually observed by the hand surgeon in all three patients and were referred to the biomechanics laboratory for gait analysis. To assess gait changes due to surgery intervention, preoperative and postoperative gait analyses were conducted. For all patients, the preoperative gait analysis was completed a week before surgery, whereas the postoperative observations were executed at 3, 6, and 12 months after surgery. During each experimental session, 34 retroflective markers (12 mm in diameter), were placed at appropriate anatomic landmarks on the body according to a standardized protocol (Kadaba et al., 1990). The patient walked at a self-selected pace in a straight line for approximately 30 feet across four force plates (Kistler^®^, Winterthur, Switzerland) embedded into the floor. The control group were also instructed to walked at a self-selected pace to establish the normal baseline for age-matched gait. Every patient served as their own internal control, data from the reference group were only used as a benchmark. Motion data were acquired using a motion capture system (Vicon^®^ Motion Systems, Oxford, United Kingdom). This study focused on sagittal plane variables of the hip, knee, and ankle joint ROM commonly used to describe human gait.

Limb rotations of the joints hip, knee and ankle were calculated from the embedded coordinate system information in VICON^®^. The Newington-Gage model was first used to define the position of the hip, knee and ankle joint centers (Vicon^®^, 2017), then joint angles corresponding to flexion-extension were computed using the Euler angles algorithm (Kadaba et al., 1990). The joint kinematic trajectories were normalized to percent gait cycle, beginning and ending with initial contact of the affected foot, and ensemble averaged over the gait cycles collected and plotted. The contralateral side (sound side), during the same gait cycle, was also plotted to track any abnormalities adapted as a compensatory strategy. Spatiotemporal measures (cadence, walking speed, stride time, stride length, single support, double support) were collected bilaterally during gait trials. Each patient served as their own control group, the data from the normal subjects were only reported for reference. Due to the uniqueness of each patient’s diagnosis and the treatment plan, preoperative and postoperative gait parameters and lower limbs kinematics data were measured and reported separately for each patient.

## Results

**FIGURE 1:**
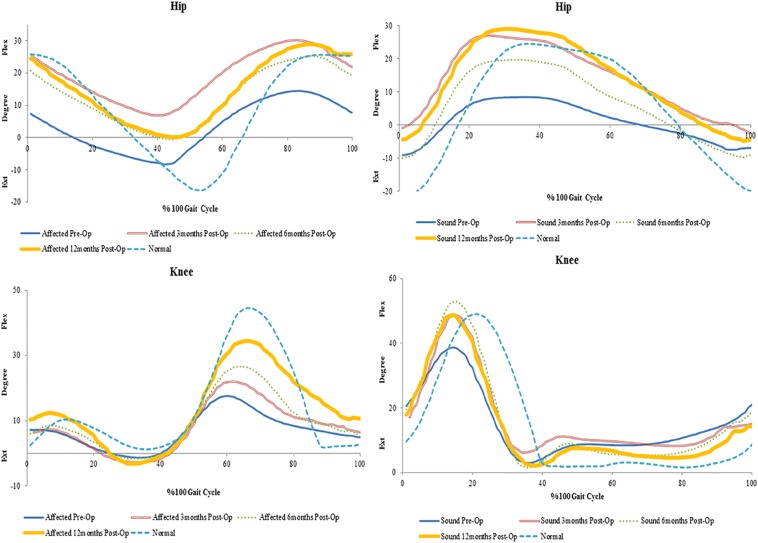
Preoperative and 3, 6, and 12 months postoperative spatiotemporal gait parameters in spastic patients (SP1–SP3) and matched normal subjects (N1–N3).

All clinical assessments presented in this study were made preoperatively at 3, 6, and 12 months postoperatively. Preoperative and postoperative gait parameters were measured and plotted longitudinally. The postoperative clinical assessments of the hand function included physical examination, SW sensory testing (Cunha et al., 2019; Dinçer and Samut, 2019) and muscles strength testing using the Manual Muscle Strength chart (Brandsma et al., 1995; Lu et al., 2008). Kinematics results of the hip, knee, and ankle in each patient varied; however, spatiotemporal measurements showed overall improvements overtime in all patients (Figure 1).

### SP1

**FIGURE 2:**
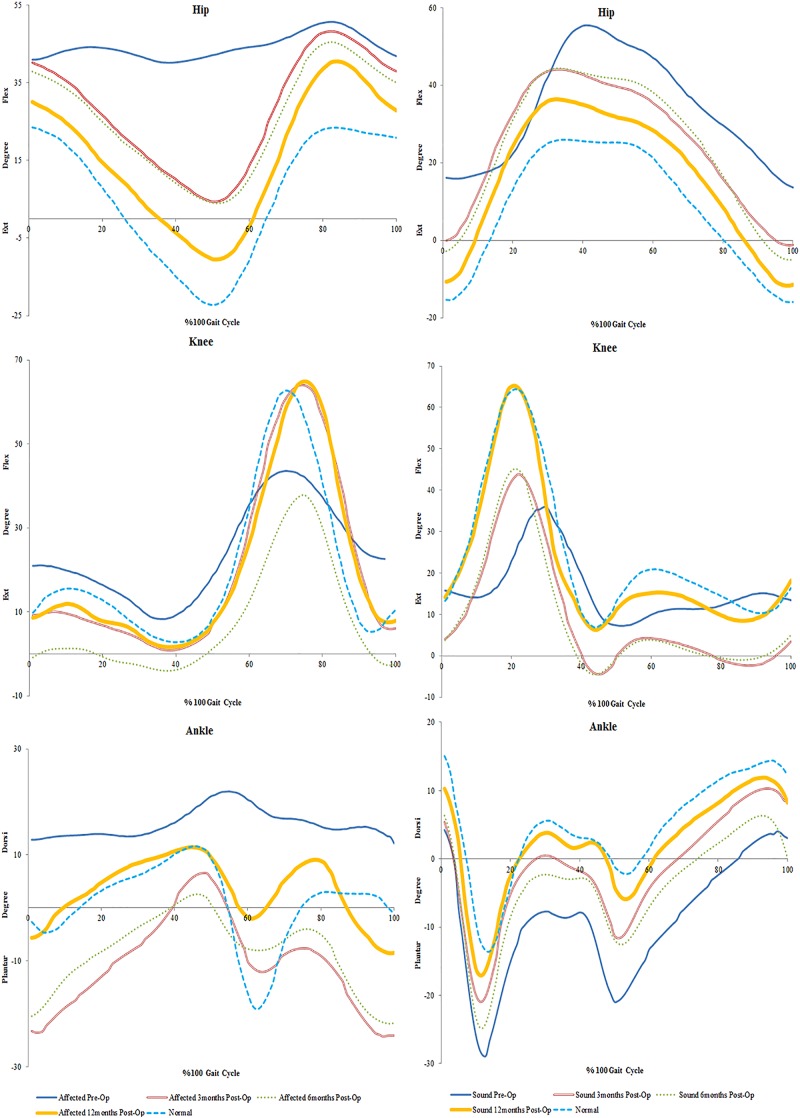
Mean trends of knee flexion-extension in the affected and sound side during gait cycle in spastic patient 1.

In SP1, sensation was regained, and pain was diminished. Improvements were seen in the overall function of his elbow, wrist and fingers. Incremental increases in peak knee flexion on both the affected (21%) and sound side (16%) were seen (Figure 2). No major changes were seen in peak hip and ankle on both sides (<5%) (Table 1). His cadence increased by 30.73 points and his walking speed almost quadrupled. This resulted in a decreased stride time (55%) and increased stride length (82%). Moreover, there was also an increase in single support and decrease in double support (9%) (Figure 1).

**TABLE 1:** Range of motion of the hip, knee and ankle joints in SP1 and degree change from preoperative to 12 months postoperative values.

**ROM (°)**	**Pre-Op**		**3 months Post-Op**		**6 months Post-Op**		**12 months Post-Op**		Pre to 12 months*		**N1 Mean (SD)**	
						
	**Affected**	**Sound**	**Affected**	**Sound**	**Affected**	**Sound**	**Affected**	**Sound**	**Affected**	**Sound**	**Right**	**Left**
Peak hip flexion	49.36	46.31	40.60	44.28	59.24	43.39	46.87	43.39	3	3	24.45 (1.05)	27.05 (1.91)
Peak hip extension	29.28	35.26	30.33	27.13	37.84	34.02	28.35	30.21	1	5	−19.15(2.02)	−14.41(1.06)
Peak knee flexion	25.79	30.83	37.94	37.63	43.89	33.11	46.63	46.61	21	16	66.64 (0.80)	67.26 (1.71)
Peak knee extension	24.23	20.27	23.44	19.29	4.58	21.30	25.24	22.09	1	2	7.33 (1.74)	9.82 (0.74)
Peak ankle dorsiflexion	7.73	18.07	25.10	20.54	22.60	19.95	25.06	19.93	17	2	25.34 (0.56)	18.52 (1.06)
Peak ankle plantarflexion	–1.33	–0.29	7.44	6.74	5.01	4.92	10.43	5.57	12	6	−8.67(1.44)	−4.91(1.92)

SP1, spastic patient 1; N1, matched normal subject with spastic patient 1. *Degree change from preoperative to 12 months postoperative values. Mean (SD) values from matched normal subject N1 were reported as reference.

### SP2

**FIGURE 3:**
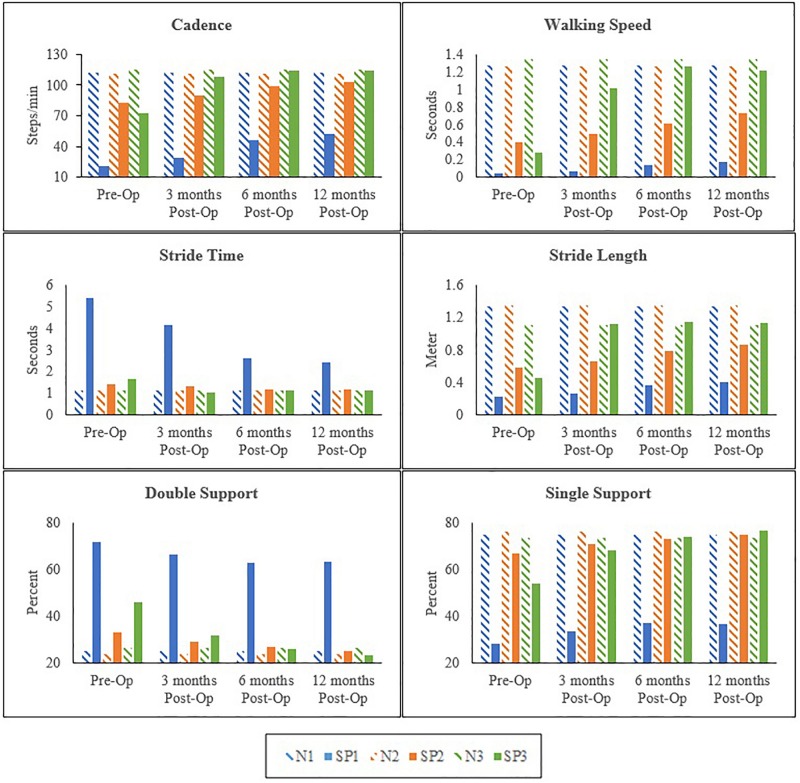
Mean trends of hip and knee flexion-extension in the affected and sound side during gait cycle in spastic patient 2.

Sensation was regained in SP2 and pain was diminished. The patient also had complete recovery in hand strength with improvements in hand function. Increases were observed on the affected side in peak hip flexion (15%) and on the sound side (21%). Peak knee flexion was also increased in the affected (17%) and sound (10%) sides (Figure 3 and Table 2). No major changes were seen in the ankle on both sides (<5%) (Table 2). There was an increase in her cadence (24%), walking speed (83%) and stride length (47%). Additionally, a decrease in stride time (18%) and double support (8%) were observed (Figure 1).

**TABLE 2:** Range of motion of the hip, knee and ankle joints in SP2 and degree change from preoperative to 12 months postoperative values.

**ROM (°)**	**Pre-Op**		**3 months Post-Op**		**6 months Post-Op**		**12 months Post-Op**		Pre to 12 months*		**N2 Mean (SD)**	
						
	**Affected**	**Sound**	**Affected**	**Sound**	**Affected**	**Sound**	**Affected**	**Sound**	**Affected**	**Sound**	**Right**	**Left**
Peak hip flexion	14.47	8.35	30.21	26.91	25.27	19.60	28.96	28.93	15	21	25.46 (1.14)	25.82 (1.29)
Peak hip extension	–8.33	–9.19	6.88	–2.53	–0.49	–10.09	0.14	–4.92	8	5	−21.63(0.55)	−16.34(0.40)
Peak knee flexion	17.67	38.67	22.04	48.76	26.63	52.89	34.54	48.65	17	10	49.03 (1.17)	44.57 (1.86)
Peak knee extension	–1.29	2.91	–2.66	6.14	–1.61	1.50	–3.15	2.13	2	1	1.55 (0.39)	1.27 (0.56)
Peak ankle dorsiflexion	8.64	7.69	16.17	4.58	9.92	15.85	12.32	11.64	4	4	19.37 (0.28)	15.66 (0.36)
Peak ankle plantarflexion	–21.06	–9.64	–8.40	–15.33	–20.43	–11.64	–19.31	–11.23	2	2	−10.71(0.72)	−15.37(0.093)

SP2, spastic patient 2; N2, matched normal subject with spastic patient 2. *Degree change from preoperative to 12 months postoperative values. Mean (SD) values from matched normal subject N2 were reported as reference.

### SP3

**FIGURE 4:**
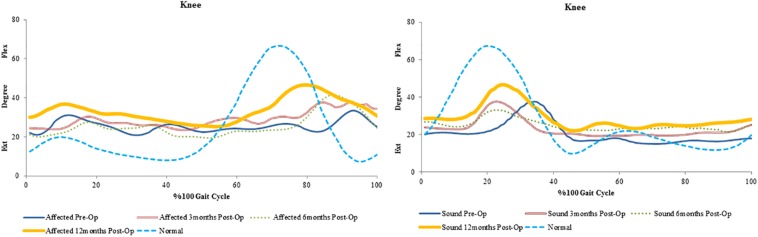
Mean trends of hip, knee and ankle flexion-extension in the affected and sound side during gait cycle in spastic patient 3.

The most noticeable changes were seen in SP3 across all his assessments. Pain was diminished and he had improved hand strength and function. A decrease of hip extension on the affected side (51%) and the sound side (25%) were observed. Knee flexion increased on both sides (affected 21% and sound 29%). Changes in the ankle were seen only in plantarflexion, with a decrease on the affected side (21%) and an increase on the sound side (12%) (Figure 4 and Table 3). His cadence increased (58%) resulting in a fivefold increase in walking speed. Stride time decreased (32%) and stride length almost tripled. Also, an increase in single support and decrease in double support (23%) were seen (Figure 1).

**TABLE 3:** Range of motion of the hip, knee and ankle joints in SP3 and degree change from preoperative to 12 months postoperative values.

**ROM (°)**	**Pre-Op**		**3 months Post-Op**		**6 months Post-Op**		**12 months Post-Op**		Pre to 12 months*		N3 Mean (SD)*	
						
	**Affected**	**Sound**	**Affected**	**Sound**	**Affected**	**Sound**	**Affected**	**Sound**	**Affected**	**Sound**	**Right**	**Left**
Peak hip flexion	48.20	55.53	48.26	44.13	45.51	44.45	43.23	36.41	5	19	23.52 (2.25)	25.95 (1.17)
Peak hip extension	40.18	13.65	4.35	–1.26	3.97	–5.07	–10.53	–11.73	51	25	−22.24 (Thevenin-Lemoine et al., 2013)	−15.99(0.40)
Peak knee flexion	43.57	35.96	64	43.87	37.81	45.15	64.88	65.23	21	29	62.70 (0.74)	64.41 (1.17)
Peak knee extension	7.28	7.23	0.83	–4.50	–4.06	–4.56	2.90	6.25	4	1	2.79 (0.78)	6.82 (1.26)
Peak ankle dorsiflexion	17.40	5.22	6.60	10.29	–8.19	–4.28	12.77	9.88	5	5	11.65 (0.71)	15.03 (0.51)
Peak ankle plantarflexion	12.20	–28.79	–24.27	–20.83	–21.86	–24.80	–8.54	–16.97	21	12	−19 (Thevenin-Lemoine et al., 2013)	−13.56(0.36)

SP3, spastic patient 3; N3, matched normal subject with spastic patient 3. *Degree change from preoperative to 12 months postoperative values. Mean (SD) values from matched normal subject N3 were reported as reference.

## Discussion

This is the first study assessing the effect of upper limb surgical intervention on gait in spastic patients. The assessment was conducted through measurements of gait parameters and lower limb joint kinematics.

Surgical planning was based on spasticity, contractures and flexibility. All surgeries were performed in efforts to stabilize and improve the position of the hand, eliminate pain, and attain as much function as possible in the hand, wrist, and fingers. Common surgical procedures for spastic upper extremity includes flexor slide, ulnar nerve release, carpal tunnel release, ulnar nerve neurectomy at the wrist, fractional lengthening of the pronator teres and wrist fusion (Fasano et al., 1978; Waters, 1978; Lazorthes et al., 2002; Maarrawi et al., 2006). The flexor slide and ulnar nerve release procedures were performed in all patients mainly to control or diminish the stretch reflexes and relieve the spasm and/or pain associated with the wrist flexion deformities. The goal was to promote better muscle balance around the wrist and hand, thus enhancing extensor functionality (Dietz, 2002). Releasing the entrapment of nerves allows proper nerve conduction which helps improve sensation, perception of space, and motion of limb in space (Lazorthes et al., 2002). This could explain the restoration of sensation in SP1 and SP2 and the improved balance seen in both; which was evident in the improvements of spatiotemporal parameters related to dynamic balance during walking (e.g., the reduction in double support phase duration that is linked to an increase of stability during walking).

Preoperative peak values of hip flexion/extension values in SP1 and SP3 on both affected and sound side were higher than the corresponding values in matched controls. For SP2, the peak values of hip flexion/extension were higher and lower than the corresponding values of the matched control, respectively. After 12 months post-surgery, improvements to normalcy in peak values of hip flexion/extension were seen on both sides in SP3 and on the sound side in SP2. Our preoperative results are in agreement with previous studies reporting ranges of hip flexion and/or extension in spastic patients being larger or smaller than those observed in normal subjects (Richards and Knutsson, 1974; Pinzur et al., 1987; Burdett et al., 1988). Hip flexor muscles (i.e., iliopsoas, rectus femoris, tensor fasciae latae, sartorius) play a fundamental role in hip extension and flexion during gait (Olney and Richards, 1996), and their impairment can result in abnormal hip motion (Simonsen et al., 2012). Hence, the improvements in hip motion observed postoperatively would suggest a better neuromuscular activation of the hip flexor muscles.

An increase in peak knee flexion at 12 months was seen in all patients. The reduction in peak knee flexion seen preoperatively is also known to be a common gait deviation in spastic individuals (Moore et al., 1993; Moseley et al., 1993). Several possible causes exist for the loss of knee flexion. Clinical observations suggest that the inability to sufficiently activate the knee flexor is the most common cause of decreased peak knee flexion during spastic gait (Winters et al., 1987). Moreover, over activity of the rectus femoris is also considered one of the factors contributing to a decreased knee flexion (Waters et al., 1979; Perry, 1987). This increase from pre-surgery values could suggest proper activation of the muscles involved in knee flexion during gait.

It was also found that there was an overall improvement in peak ankle dorsi/plantar flexion only in SP3 at 12 months. When a decrease of dorsiflexion is evident in gait, it is likely due to an inability to generate a sufficiently large dorsiflexion muscle moment (Dimitrijević et al., 1981). Similarly, and perhaps even more commonly, a reduced dorsiflexion may be caused by an increased muscle moment attributable either to adaptive shortening or excessive activation of the plantarflexion muscles (Moore et al., 1993). This could imply that the increase seen at 12 months was a result of improved ankle flexor functions.

In the present case studies, there is a marked heterogeneity in the clinical presentation of the participating patients as demonstrated by differences in age, disease onset and diagnosis. However, studies show that the neurobiological background for the development of spasticity is similar between patients with different causes of brain damage (Sunnerhagen et al., 2018; Gschwind et al., 2019) and, therefore, same treatment goals can be applied to different diagnosis of brain damage. Several key factors impact the extent of gait recovery after a brain injury; such as age and the severity of spasticity due to brain injury (Teasdale et al., 1979; Katz et al., 2004; Katz-Leurer et al., 2009). The results of this study indicated that the youngest patient, SP3, had the most substantial recovery. This is in agreement with previous studies (Teasdale et al., 1979; Authier et al., 1995) suggesting that there is a plausible effect of age and functional recovery, as youth plays a prominent factor in neuroplasticity following brain injury (Uswatte and Taub, 2010; Rutović et al., 2019). Additionally, other studies have shown that, depending on the severity of spasticity, some patients might demonstrate significant gait recovery levels (e.g., increased walking velocity) while other might show minimal improvements (Knutsson and Richards, 1979; Kuhtz-Buschbeck et al., 2003; Watson and Hitchcock, 2004; Tilson et al., 2010; Jang and Kwon, 2016; Marque et al., 2019), as observed postoperatively in SP1. Even though the joint kinematic parameters of the lower limbs in SP1 didn’t show outstanding improvements over time, at 12 months postoperatively the patient was able to ambulate comfortably with a cane; no longer relying on a wheelchair. Post-stroke patients often use wheelchairs when they are not able to put any weight on their lower limbs due to the severity of the disability (Zorowitz, 2005; Mikolajewska, 2012). Using a cane, postoperatively, demonstrated a large improvement in achieving gait independence which was evident in the improvements of SP1’s gait parameters overtime.

Spastic gait is characterized by slow cadence, reduced walking time, increased stride time and double support (Roth et al., 1997) all of which was seen in all three patients prior to the surgery. At 12 months, an increase in cadence, walking speed, stride length and a decrease in stride time were seen in all. A decrease in double support and an increase in single support were also observed in all patients. Walking time is an indicator of overall gait performance and it has been associated with gait parameters such as cadence, stride length and double support (Brandstater et al., 1983; Frenkel-Toledo et al., 2005; Ford et al., 2007). Therefore, an increase in walking speed improves these parameters. Overall, the recovery in gait parameters seen in this study suggests improved balance and muscle strength (Turnbull et al., 1995; Chen et al., 2005) in patients.

## Conclusion

We presented a collection of case studies. The number of patients investigated might be limited, and their clinical presentation might be heterogeneous. Nevertheless, the results we report for each case consistently suggest that upper limb surgery has the ability to improve gait spatiotemporal parameters and lower limb joints ROM. Future studies on a greater number of spastic patients are needed to further support our preliminary findings that releasing upper limb spasticity improves gait performance in spastic patients over time.

## Ethics Statement

The studies involving human participants were reviewed and approved by the Institutional Review Board at the University of Miami. The patients/participants provided their written informed consent to participate in this study.

## Author Contributions

NA, SA, and EO contributed to the conception and design of the study. NA ran the experiments, collected data, and wrote the first draft of the manuscript. SA supervised the study. SA, FT, and EO reviewed and revised the results. All authors contributed to the manuscript revision, read, and approved the submitted version.

## Conflict of Interest

The authors declare that the research was conducted in the absence of any commercial or financial relationships that could be construed as a potential conflict of interest.
